# Functional Verification of the Soybean Pseudo-Response Factor *GmPRR7b* and Regulation of Its Rhythmic Expression

**DOI:** 10.3390/ijms26062446

**Published:** 2025-03-09

**Authors:** Ziye Song, Jia Liu, Xueyan Qian, Zhengjun Xia, Bo Wang, Nianxi Liu, Zhigang Yi, Zhi Li, Zhimin Dong, Chunbao Zhang, Bo Zhang, Million Tadege, Yingshan Dong, Yuqiu Li

**Affiliations:** 1 Jilin Academy Agricultural of Science (Northeast Agricultural Research Center of China), Changchun 130033, China; skysziye@163.com (Z.S.); lj_hope0459@aliyun.com (J.L.); xueyanqian@126.com (X.Q.); bowang1122@126.com (B.W.); lnx69@126.com (N.L.); yizhigang0504@126.com (Z.Y.); lizhi527@126.com (Z.L.); dongzhimin2005@126.com (Z.D.); cbzhang@cjaas.com (C.Z.); 2 Northeast Institute of Geography and Agroecology, Chinese Academy of Sciences, Harbin 150081, China; xiazhj@iga.ac.cn; 3 School of Plant and Environmental Sciences, Virginia Polytechnic Institute and State University, Blacksburg, VI 24061, USA; bozhang@vt.edu; 4 Institute of Agricultural Biosciences, Oklahoma State University, Stillwater, OK 74078, USA; million.tadege@okstate.edu

**Keywords:** soybean, *PRR*, rhythmic expression, interaction

## Abstract

The pseudo response regulator (*PRR*) gene is an important component of the core oscillator involved in plant circadian rhythms and plays an important role in regulating plant growth and development and stress responses. In this study, we investigated the function of *GmPRR7b* by overexpression and gene editing approaches. It was found that *GmPRR7b* plays a role in delaying flowering. While *GmPRR7b* overexpressing plants showed significantly delayed flowering compared to untransformed WT, *GmPRR7b* edited plants flowered earlier than the control WT. On the basis of previous research results and bioinformatics analysis, we re-identified 14 soybean *PRR* genes and analysed their rhythmic expression. Based on the rhythmic expression pattern, we found that GmPRR5/9a and GmPRR5/9b interacted with GmPRR7b by yeast two-hybrid and bimolecular fluorescence complementation (BiFC) experiments. Combined with the expression regulatory networks of the *GmPRR7b*, we inferred a possible regulatory mechanism by which *GmPRR7b* affects flowering through quit rhythm expression. These research elements provide valuable references for understanding growth, development, and circadian regulation in soybean.

## 1. Introduction

Following the rhythmic phenomenon of the alternation of day and night caused by the rotation of the Earth, plants have a self-regulating mechanism with an approximate 24 h rhythm [1,2]. This endogenous rhythmic regulatory mechanism in plants is called the biological clock or circadian rhythm [3]. The plant circadian system regulates almost all growth and developmental and metabolic processes, such as flowering, leaf movement, and hormone signalling [4,5,6]. The clock consists of three parts: an input pathway, a core oscillator, and an output pathway [7]. The core oscillator is a transcriptional–translational feedback loop consisting mainly of two MYB proteins, LHY and CCA1, and the family of pseudo-response regulators (*PRRs*) [8].

The first identification of *PRRs* was in the model plant, *Arabidopsis* (*Arabidopsis thaliana* (L.) Heynh.) [9]. Scientists discovered that *PRR* genes exhibit robust rhythmicity with an expression cycle of approximately 24 h [9,10]. The transcript levels of *AtPRR9*/*AtPRR7*/*AtPRR5*/*AtPRR3*/*AtPRR1* in *Arabidopsis* accumulate sequentially from morning to evening, reaching a peak within 2–3 h [9,10,11,12,13,14]. Furthermore, *AtPRRs* have been demonstrated to regulate diverse processes, including photoperiodic regulation of flowering, hypocotyl growth, and seed germination [15,16]. In addition to these functions, *AtPRRs* have been shown to respond positively to drought stress and cold stress [17,18]. The present research on *PRRs* in *Arabidopsis* is predominantly centred on the photoperiodic regulatory pathway of this species [18]. The “*GI-CO-FT*” pathway, which has been extensively studied, is a notable example of this regulatory mechanism, with *PRRs* playing a pivotal role in transmitting rhythmic signals [19,20,21,22].

Because of these interesting rhythmic expression patterns, *PRRs* have quickly become a popular research focus on plants. For example, five *PRR* genes (*OsPRR1*, *OsPRR37*, *OsPRR73*, *OsPRR59*, and *OsPRR95*) have also been identified in rice (*Oryza sativa* L.) and shown to be involved in the regulation of the biological clock [23,24,25,26,27]. Thirteen *PRR* genes have been identified in tomato (*Solanum lycopersicum* L.) [28,29], and Wang et al. [30] identified a total of forty-four *PRR* genes in four species of cotton (*Gossypium hirsutum* Linn.). These studies collectively indicate that *PRRs* are rhythmically expressed.

Research on *PRRs* in soybean (*Glycine max* (L.) Merr.) has progressed more slowly compared to model plants such as *Arabidopsis* and rice. Zhang et al. [31] used a homologous comparison to obtain five homologous genes of *PRR5/9*, four homologous genes of *PRR7*, and four homologous genes of *TOC1* (*PRR1*) to study circadian rhythm genes in soybean. This is the first time that *PRR* genes have been identified in soybeans. Subsequently, Li et al. [32] obtained two homologous genes of *APRR3*, *GmPRR3a*, and *GmPRR3b*, from a recombinant inbred line population of wild soybean (*Glycine soja* Siebold & Zucc.) and soybean. Li et al. [33] also identified a gene homologous to *PRR7* in a population of local and cultivated soybean varieties. Wang et al. [34] also identified *GmPRR37* in a population of recombinant inbred lines and developed a mutant suitable for planting in high-latitude areas or under multiple cropping conditions. Similarly, Li et al. [35] also obtained a *GmPRR3b* gene through a genome-wide association study and found that overexpression of a haplotype of this gene increased the number of main stem nodes and yield. Lu et al. [36] identified two *PRR3* homologous genes, *Tof11* and *Tof12*, and found that these two genes can directly bind to the *LHY* promoter to inhibit its transcription, which adds to the mechanism of photoperiod regulation of flowering in soybeans.

Based on previous laboratory studies [33], this study further studied the function of the identified *GmPRR7b* gene. Then, by analysing the rhythmic expression of its gene family members, we try to determine the potential interaction proteins of the target gene. These interactions are verified by real-time quantitative PCR (qRT-PCR), yeast two-hybrid detection, and BiFC. It is expected to provide additional evidence for elucidating the role of circadian rhythm signals in regulating soybean photoperiod response.

## 2. Results

### 2.1. Phenotypic Characterisation of the Glyma.12G073900

**Figure 1:**
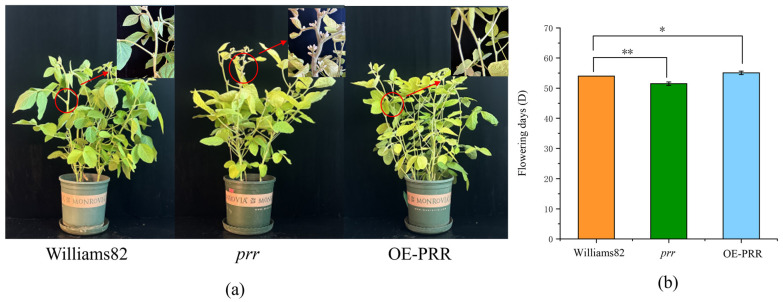
Mutant, over-expression, and control plants and flowering time. (**a**), Mutant, overexpression, and control plants; (**b**) flowering time. * Statistical significance at 0.05 level; ** Statistical significance at 0.01 level.

We obtained the *Glyma.12G073900* in our preliminary work by obtaining a QTL related to flowering and screening it by localization [33]. To verify the function of *Glyma.12G073900*, transgentic lines overexpressing (OE-PRR) and gene-edited lines (*prr*) were obtained by transgenic technology (Figure S1, Table S1). We found that OE-PRR bloomed significantly later than the wild type, while the *prr* bloomed significantly earlier than the wild type (Figure 1), indicating that *Glyma.12G073900* overexpression delayed flowering, while *Glyma.12G073900* loss-of-function accelerated flowering time. This result suggests that *Glyma.12G073900* functions as a floral repressor in soybean under standard growth conditions.

### 2.2. Identification and Rhythmic Expression of Gene Family Members

Through a previous study by Li et al. [33], we found that *Glyma.12G073900* belongs to the *PRR* gene family, so we re-identified the *PRR* gene family members. We searched the soybean genome data and obtained 12 *PRR* genes that contain both conserved structural domains of the *PRR* gene family. So *Glyma.12G073900* is *GmPRR7b*. Although *GmPRR7a* and *GmPRR7b* do not contain the CCT domain, previous studies [32,33,34,35,36,37,38] have demonstrated their involvement in flowering, and we still consider them to be part of the *PRR* gene family. In total, we determined that there are 14 members of the PRR gene family in soybean (Table S2).

**Figure 2:**
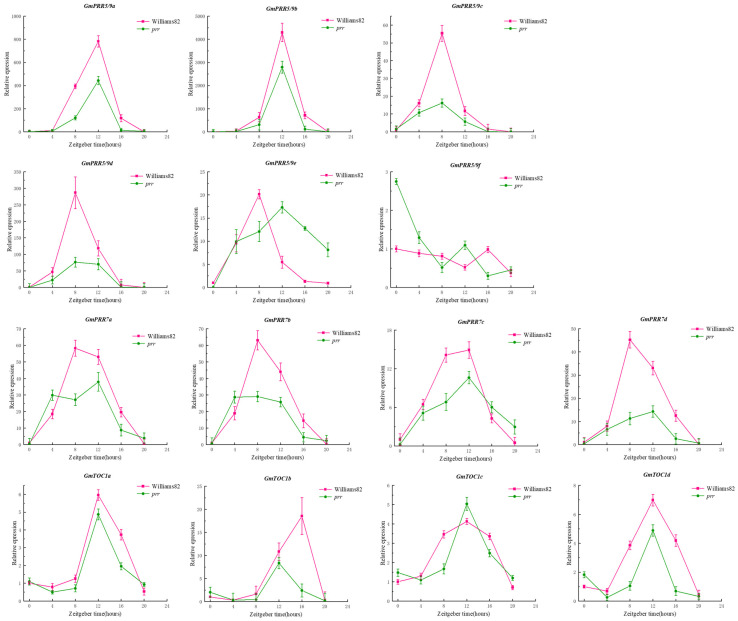
Rhythmic expression of gene family members.

Due to the rhythmic expression characteristics of the *PRR* gene family, we tried to find the rhythmic expression pattern of *GmPRRs* as follows (Figure 2): We found that members of the gene family are characterised by rhythmic expression, and *GmPRR5/9s* appear to be classified into three groups based on the timing of expression, in Williams 82. *GmPRR5/9c*, *GmPRR5/9d*, *GmPRR5/9e*, *GmPRR7b*, and *GmPRR7d* were the first to reach peak expression at ZT8, then *GmPRR7a*; and *GmPRR7c* showed peak expression at both ZT8 and ZT12. Other *PRR* genes (*GmPRR5/9a*, *GmPRR5/9b*, *GmTOC1a*, *GmTOC1c*, *GmTOC1d*) basically reached peak expression at ZT12, and *GmPRR5/9f* and *GmTOC1b* were the latest, reaching peak expression at ZT16. In *prr* material, *GmPRR5/9f* was the first to reach peak expression at ZT0; *GmPRR7a* and *GmPRR7b* attained high expression at ZT4, followed by *GmPRR5/9c*, reaching peak expression at ZT10, and *GmPRR5/9d* reached a high expression, from ZT8 to ZT12, while the remaining genes reached peak expression at ZT12. These results suggest that *PRR* genes not only exhibit rhythmic expression but also may interact with one another within the gene family to respond to the light signal [9,10,11,12,13,14].

### 2.3. Verification of the Interaction Between GmPRR5/9a, GmPRR5/9b, and GmPRR7b

**Figure 3:**
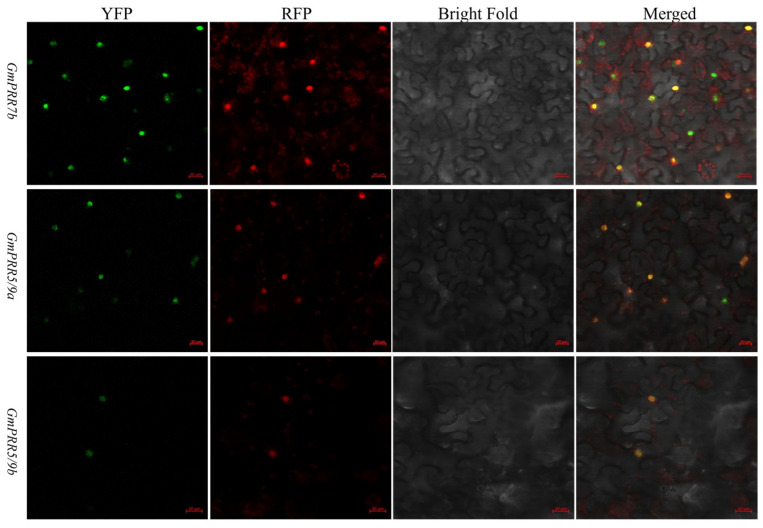
Subcellular localization. Transient expression of *pEarleygate104-GmPRR7b*, *pEarleygate104-GmPRR5/9a*, *pEarleygate104-GmPRR5/9b*, and *PC1302-RFP-PIP2* fusion proteins in tobacco; green indicates the fluorescent colour of YFP, red indicates the fluorescent colour of RFP, and yellow indicates the fluorescent colour of YFP and RFP complexed out (bar = 20 μm).

**Figure 4:**
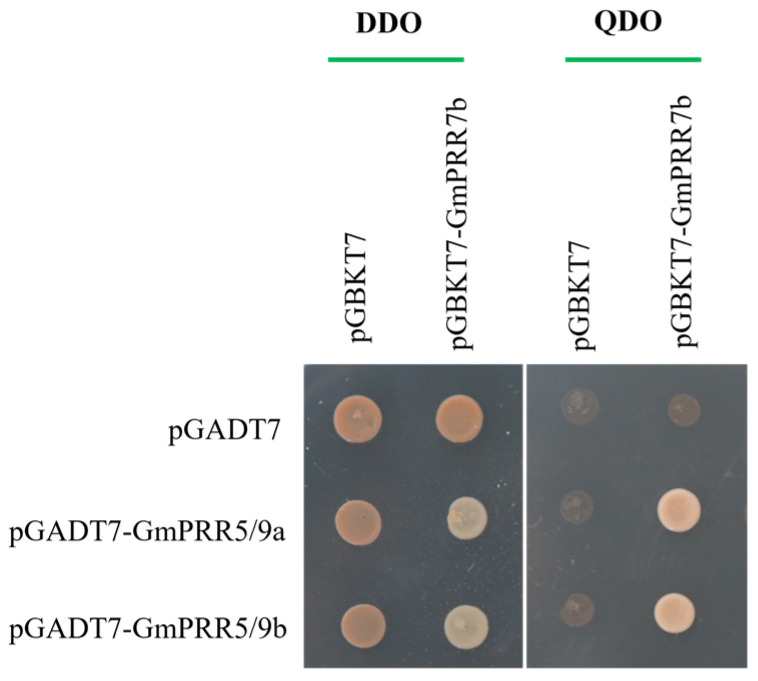
Interaction protein screening of GmPRR7b.

To test the above-stated, we selected GmPRR5/9a and GmPRR5/9b, which peaked after GmPRR7b, for yeast two-hybrid verification based on the order of expression. We first performed subcellular localization verification experiments on these three proteins (Figure 3). The results showed that all three proteins were localized in the nucleus, consistent with the previous prediction. On a high-stringency quadruple drop-out medium (QDO plate), the GmPRR5/9a and GmPRR7b plate grew white colonies, as did the GmPRR5/9b and GmPRR7b plate, indicating that both GmPRR5/9a and GmPRR5/9b interact with GmPRR7b at the protein level in yeast (Figure 4).

**Figure 5:**
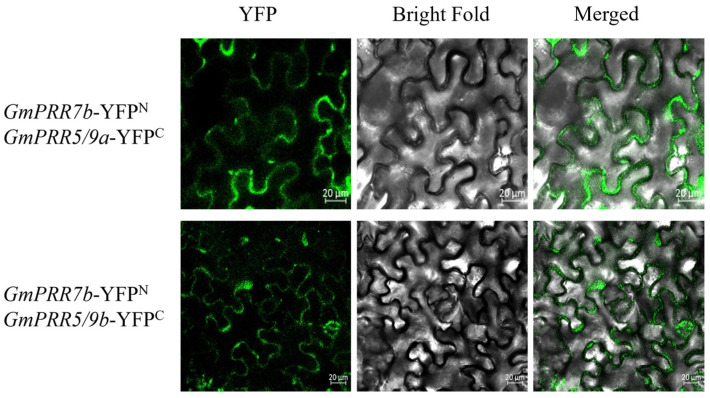
BIFC for GmPRR7b.

To further verify the results of the yeast two-hybrid assay, we performed a BiFC assay using the split YFP system to confirm the interaction in living plant cells (Figure 5). Imaging with laser confocal microscopy revealed that there is YFP fluorescence in both construct pairs, indicating that GmPRR7b interacts with both GmPRR5/9a and GmPRR5/9b.

### 2.4. Gene Regulatory Network Prediction

**Figure 6:**
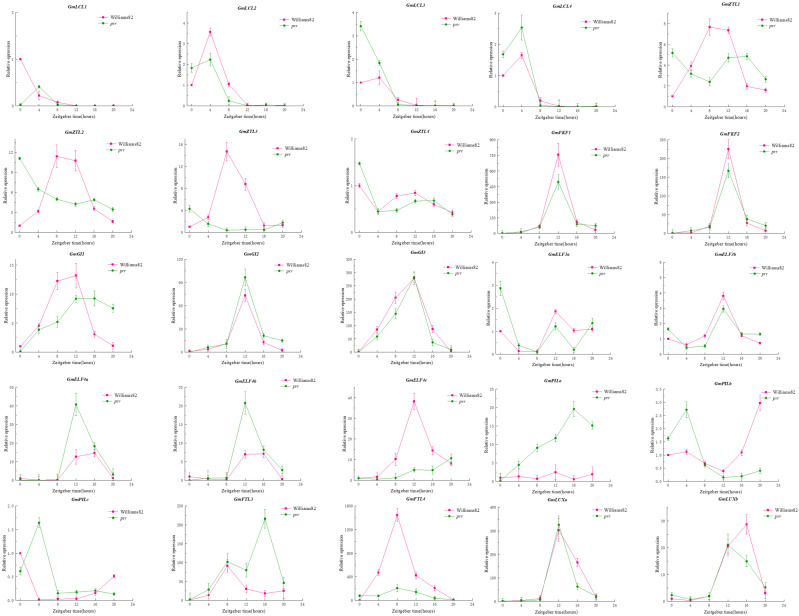
qRT-PCR validation of gene regulatory networks.

Based on the fact that circadian rhythm properties are closely linked to photoperiod [36,39] and we have demonstrated that there are interactions between gene family members, we used *prr* material to perform qRT-PCR on some of the currently known photoperiodic genes in order to be able to resolve the mechanism of GmPRR7b with the photoperiodic regulatory network (Figure 6). The results indicated that *GmZTLs* (*GmZTL1*, *GmZTL2*, *GmZTL3*), *GmELF4s* (*GmELF4a*, *GmELF4b*, *GmELF4c*), *GmPILs* (*GmPILa*, *GmPILb*, *GmPILc*), as well as *GmFIL3* and *GmFIL4* might be regulated in association with *GmPRR7b* expression.

## 3. Discussion

The two-component signal transduction system (TCS), the main mechanism of extracellular signal transduction, consists of a histidine protein kinase (HK) and a response regulator (RR). The response regulators (RR) are very similar to pseudo-response regulators [40]. In typical TCSs, once the HK senses a stimulus, the His protein kinase self-phosphorylates its conserved His residue to regulate its own signal, transferring the phosphate group to the conserved Asp residue in the RR acceptor domain to stimulate activity and respond accordingly [41]. The RR has an N-terminal receptor domain and a C-terminal output domain. The classical N-terminal receptor domain has a negatively charged amino acid surrounded by an N-terminal aspartic acid (D), a central aspartic acid site (D) that receives a phosphate group, and a C-terminal lysine (K), called the DDK sequence. Several DDK variants that are very similar to the classical DDK sequence have been found in *Arabidopsis* [42]. In these variants, the aspartic acid at the phosphorylation acceptor site is replaced with glutamic acid, and some amino acid positions differ. This type of protein is called pseudo-response regulatory protein (PRR), while the original classical DDK sequence regulatory protein is called response regulatory protein (RR) [43]. However, because PRR still contains an Asp residue in the conserved motif, it can still be used as the final output of the two-component phosphorelay in plants. Therefore, it is speculated that PRRs may be involved in the signal transduction of the His-to-Asp phosphorelay and the regulation of circadian rhythms.

Our previous work successfully localised the GmPRR7b gene on chromosome 12 [33]. So, we verified the gene function of *GmPRR7b* by constructing overexpression lines and gene editing lines. We suggest that the *GmPRR7b* gene is a deterrent to flowering, which is consistent with the findings of Wang et al. [34] and Lu et al. [36]. This could further confirm that *GmPRR7b* could provide a new target for creating soybean materials.

In *Arabidopsis*, *PRRs* are known clock factors involved in rhythmic expression in the core oscillator located in the photoperiod-regulated flowering pathway [44,45]. We re-identified all *PRR* gene family members in soybean and screened 12 members based on the structure and characteristics of the *PRR* gene family, which is consistent with the results of Wang et al. [30] and Zhang et al. [31]. There has been a lot of evidence [32,33,34,35,36,37,38] showing that *GmPRR7a* and *GmPRR7b*, although they are missing the CCT structural domains, can regulate flowering time. Not only that, in fact, we found that the wild type of *GmPRR7b* is equipped with the CCT structural domain and exhibits late flowering. However, it is CCT-deficient in bred varieties, such as W82 and CN16, which exhibit early flowering. Therefore, we agree with previous authors [30,31] that *GmPRR7a* and *GmPRR7b* belong to the *PRR* gene family, and the rhythmic expression of the *PRR* gene family members in *prr* material shows a different expression pattern from that in W82, which reveals the characteristic of the circadian cycle, ‘A single thread can pull the whole system’. After the change in GmPRR7b, GmPRR5/9a and GmPRR5/9b are the most direct changes. Therefore, we first chose to test the interaction between these two genes, and the yeast two-hybrid and BiFC experiments showed that they interacted with each other, especially in the BiFC experiments; we found that both of them were nuclear localised. GmPRR7b, GmPRR5/9a, and GmPRR5/9b and the interaction in BiFC was shown at the cell membrane, and we believe that when circadian signals are transmitted to GmPRR7b, GmPRR5/9a, and GmPRR5/9b, which are expressed downstream, interact with GmPRR7b at the cell membrane, thus generating a signal that transmits signals and maintains circadian rhythms that occurs.

We identified numerous genes associated with soybean photoperiod through gene regulatory network prediction of *PRR* gene family members, all of which may be regulated by *GmPRR7b*. *GmLCLs* are homologous to *CCA1* and *LHY* and are first expressed in ZT0 and ZT4, in agreement with the results of Wang et al. [46] and Wu et al. [47]. *GmZTLs*, *GmFKFs*, and *GmGIs* have also all been shown to be associated with soybean flowering and photoperiod [48,49]; *GmELF3s*, *GmELF4s*, and *GmLUXs* are members of the EC of the soybean circadian complex [39,50,51]; in our results, *GmPRR7b* does not seem to have a regulatory relationship with *GmELF3s* and *GmLUXs*, but rather *GmELF4s* shows a strong regulatory relationship, so we infer that *GmPRR7b* is regulated by *GmELF4s* and then enters the EC complex, which influences the whole EC complex. Of course, these speculations need to be verified by further experiments, which is also the focus of our research direction in the future.

In summary, we propose a hypothesis that under prolonged sunlight, *CCA1* and *LHY* activate the expression of *GmPRR5/9a*, *GmPRR5/9b*, and *GmTOCs* through activation of *GmPRR5/9c*, *GmPRR5/9d*, and *GmPRR5/9e*, which then affects the expression of *GmPRR7s*, *GmPRR5/9a*, *GmPRR5/9b*, and *GmTOCs*, and that the *GmTOCs* then acts, in turn, on *CCA1* and *LHY*, forming a circadian cycle. When *GmPRR7b* was knocked down, *GmPRR5/9a* and *GmPRR5/9b*, which interact with *GmPRR7b*, showed reduced expression, which in turn affected the *GmTOC* expression, thereby increasing the expression of *CCA1* and *LHY* and affecting *GmELF4s* in the evening EC complex, which then affects the entire EC complex and inhibits *E1*, resulting in the early flowering phenotype.

While we identified other genes that may have a regulatory relationship with each other, more research is needed. Studies on plant *PRR* genes primarily focus on the *PRR* gene family of the model plant, *Arabidopsis*; however, the structure, function, and expression patterns of *PRR* genes in soybeans require further systematic investigation. Such studies will provide deeper insights into the molecular mechanism underlying the functions of *PRR* genes more comprehensively, offering a strong scientific basis for future studies on the molecular mechanism underlying soybean growth.

## 4. Materials and Methods

### 4.1. Preparation of Plant Material

After amplifying the target gene using the parent CN16 of the group as a template [33], the overexpression vector *pCAMBIA3301-GmPRR7b* and the gene editing vector *pKSE401-GmPRR7b* was constructed, and Williams 82 was used as the genetic transformation receptor. The Agrobacterium-mediated method [52] was used to transfer the overexpression vector into Williams 82. The transgenic positive strain was obtained by screening with a concentration (160 mg·L^−1^) of glufosinate and Bar test strip, verified by sequencing, and then propagated to the F_2_ generation. After the transgenic plants were genetically stable, the phenotype was identified under long-day conditions (16 h light/8 h dark, LD). The above-related materials were provided by the Jilin Academy of Agricultural Sciences (Changchun, China).

### 4.2. Identification of Gene Family Members

The genomic data of soybean (*Glycine max* Wm82.a2. v1) were obtained from Phytozome 13 (https://phytozome.jgi.doe.gov/pz/portal.html, accessed on 27 September 2022). Previous studies have shown that genes belonging to the *PRR* family contain two conserved structural domains: REC(PF00072) and CCT(PF06203) [28,30]. We downloaded the HMM model from the InterPro website (https://www.ebi.ac.uk/interpro/search/sequence/, accessed on 27 September 2022), used it to perform an HMM search, and obtained the search results. The intersection was considered, and the CDD database (https://www.ncbi.nlm.nih.gov/cdd/, accessed on 27 September 2022), SMART database (http://smart.embl.de/, accessed on 27 September 2022), and Pfam database (http://pfam.xfam.org/, accessed on 27 September 2022) were then used to further identify the conserved domains of the initially screened candidate protein sequences. Following previous studies on *PRR* gene family members [31], we adopted the naming conventions established in those articles. The reidentified *PRR* family members in this study retain their original names, while newly identified members were named according to the same rules (Table S1).

### 4.3. Rhythmic Expression of Members

Williams 82 and *prr* transgenic materials were grown at room temperature under long-day conditions (16 h light/8 h dark). After the third trifoliate compound leaf was fully expanded, leaf tissue was collected every 4 h for 24 h. Samples were stored at −80 °C [33].

### 4.4. RNA Isolation and Quantitative Real-Time PCR Analysis

The Plant RNA Extraction Kit (Trans, Beijing, China) was used to extract RNA from the plant samples, and the purity and concentration of the total RNA were determined using the Nanodrop system (Thermo Fisher Scientific, Waltham, MA, USA). According to the kit instructions, cDNA was synthesized using the Prime Script™RT Reagent Kit (Takara Bio, Beijing, China). Real-time fluorescence quantitative PCR (qRT-PCR) was performed on each cDNA template using the TB Green Mix (Takara Bio, Beijing, China). The PCR amplification conditions were as follows: 95 °C for 5 min, followed by 45 cycles of 95 °C for 10 s and 60 °C for 30 s in a 10 μL reaction mixture. Three replicates were prepared per sample, and the QuantStudio 6 Flex system (Thermo Fisher Scientific, Waltham, MA, USA) was used to carry out the reactions. This was iterated three times. Relative gene expression was calculated using the 2^−ΔΔCt^ method, with Tubllin serving as the internal reference gene (Table S3).

### 4.5. Validation of Gene Regulatory Networks

Expression validation was conducted as described in Section 4.3 and Section 4.4.

### 4.6. Subcellular Localization and Experimental Validation of Interacting Proteins

The target gene vector *pEarleygate104-GmPRR7b* was constructed for subcellular localization. The marker used was *PC1302-RFP-PIP2*. Subcellular localization was analysed by transient expression in tobacco leaf epidermal cells, and results were observed using confocal microscopy, following the methods described by Zhu et al. [53].

The target gene CDS sequence was linked to *pGADT7* vector, and GmPRR7b was ligated to *pGBKT7* vector for yeast two-hybrid interactions validation, and the specific experimental steps were referred to Haobo He [54].

The successful gene verified by yeast two-hybrid experiment was ligated to *pSITE-C-EYEP* vector, and *GmPRR7b* was ligated to *pSITE-N-EYFP* vector, and the results of yeast two-hybrid experiments were verified by tobacco transformation using laser confocal microscopy, and the specific experimental steps were referred to Hongxia Dong [55].

The above-related materials were provided by the Jilin Academy of Agricultural Sciences (Changchun, China).

## Data Availability

Further inquiries on data resources can be directed to the first author.

## Supplementary Materials

The supporting information can be downloaded at https://www.mdpi.com/article/10.3390/ijms26062446/s1.
