# Supplementary material for: Functional Verification of the Soybean Pseudo-Response Factor GmPRR7b and Regulation of Its Rhythmic Expression
Source: Int J Mol Sci. 2025 Mar 9;26(6):2446. doi: 10.3390/ijms26062446 (PMC11942516; doi:10.3390/ijms26062446)
Supplement: Supplementary file 1 [file ijms-26-02446-s001.zip › ijms-3464018-supplementary.pdf]

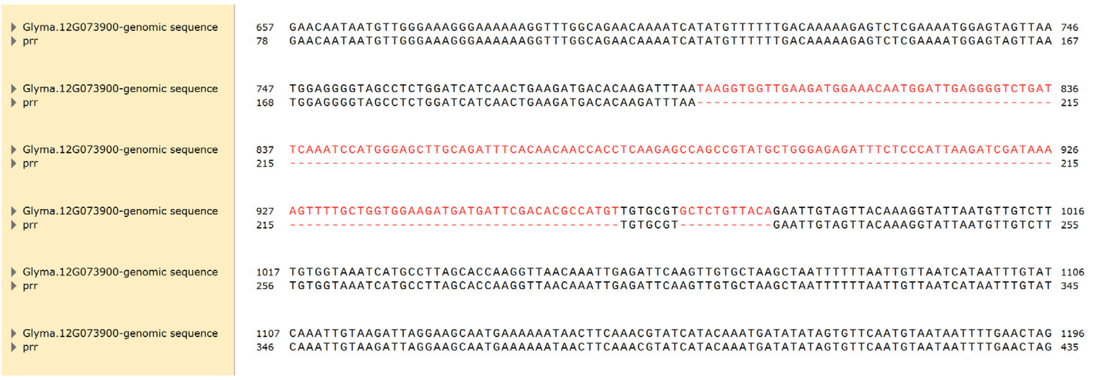

Figure S1. Schematic representation of the mutation site of the *prr* mutant

Table S1 Sequence of sgRNA

sgRNA	Sequence
sgRNA1	TGACACAAGATTTAATAAGGTGG
sgRNA2	CCATGTTGTGCGTGCTCTGTTAC

Table S2 Gene Nomenclature Control List

Our Name	Our ID	Old Name*	Old ID*
GmTOC1a	Glyma.04G166300	GmTOC1a	Glyma.04G166300
GmTOC1b	Glyma.06G196200	GmTOC1b	Glyma.06G196200
GmTOC1c	Glyma.17G102200	GmTOC1c	Glyma.17G102200
GmTOC1d	Glyma.05G025000	GmTOC1d	Glyma.05G025000
GmPRR7a	Glyma.U034500	GmPRR7a	Glyma.U034500
GmPRR7b	Glyma.12G073900	GmPRR7b	Glyma.12G073900
GmPRR7c	Glyma.13G135900	GmPRR7c	Glyma.13G135900
GmPRR7d	Glyma.10G048100	GmPRR7d	Glyma.10G048100
GmPRR5/9a	Glyma.06G136600	GmPRR5/9a	Glyma.06G136600
GmPRR5/9b	Glyma.04G228300	GmPRR5/9b	Glyma.04G228300
GmPRR5/9c	Glyma.07G049400	GmPRR5/9c	Glyma.07G049400
GmPRR5/9d	Glyma.19G260400	GmPRR5/9d	Glyma.19G260400
GmPRR5/9e	Glyma.03G261300	GmPRR5/9e	Glyma.03G261300
GmPRR5/9f	Glyma.16G018000		

* Zhang, S.R.; Wang, H.; Wang, Z.Y.; Ren, Y.; Niu, L.F. Photoperiodism dynamics during the domestication and improvement of soybea. Science China Life Sciences, 2017.

Table S3. List of primers for qRT-PCR

Primers id	sequences
GmPRR1-F	GCAGAATCAGGTGTCCATCC
GmPRR1-R	CATCACTTCTGCAGCCCATG
GmPRR2-F	TACGGGAAAATGGAGAGGCA
GmPRR2-R	TCTTGGATGCATCACACCCT

GmPRR3-F	ACATGCAGAAGATCGAGGACA
GmPRR3-R	GGTCTTCATGCTTTCCCTCTG
GmPRR4-F	GTAGTCATGGTTCTGGGCCT
GmPRR4-R	ACCAATGCTGCTTCTCTCCT
GmPRR5-F	CATCTCACGCATCGCTACTG
GmPRR5-R	GACTTGGCATTGCTGGTGAA
GmPRR6-F	TGGATTGGTGGATGGAGACC
GmPRR6-R	GGTGCCTTGCCATCAAATCA
GmPRR7-F	CGCAAGGCATGATTCTCCTG
GmPRR7-R	TGACTGGAGCGATGAGGATC
GmPRR8-F	CTTGCGGCAATCCTCCTAAC
GmPRR8-R	AGTCCAGAGCTGCAGAATCT
GmPRR9-F	GATGGAAATGCAGCGGAGAG
GmPRR9-R	AATGCAAGCCGTCCTTCATC
GmPRR10-F	TTTGACAAGAAGCCGGAGTC
GmPRR10-R	ATCCTTGTTCAGAGCCTCCT
GmPRR11-F	GCTCCAGACTTCCATCTCCA
GmPRR11-R	CAACTAGCACTTCAACCGCA
GmPRR13-F	CCTCCTCATGCGAATGGGAT
GmPRR13-R	CTTTCAGCGAGCCGTTTTCT
GmPRR14-F	AGACACATGACTCTCCTGCT
GmPRR14-R	CTGCTTCTCGTTGGCTAGTG
GmLCL1-F	GGCTTGCCTTTCAGGCTCTA
GmLCL1-R	AGCAGTCCCTCCTCCTCATT
GmLCL2-F	GGCTTGGAGAAAGAACCCCT
GmLCL2-R	ATGAACAGGGTGCCTCTTGG
GmLCL3-F	AATAGGAGGGGTTAACGGGC
GmLCL3-R	TACGGCCAAAATGTAGCAGC
GmLCL4-F	CTTTTGCCTGTTCTCCTGCA
GmLCL4-R	GGAATCTAGGTGCTCGGGAA
GmZTL1-F	AGAGAAGGCATCCGTTGGTG
GmZTL1-R	CCCGGAACAGGACCAAGATC
GmZTL2-F	CACGTCCAGGTAAGCTCTCC
GmZTL2-R	GATCTGGGAAGTGGAGGTGC
GmZTL3-F	GGAGGAATGGATGCTAAG
GmZTL3-R	ACCAATCAGAGAATCACC
GmZTL4-F	GGGATCGCAATGTTACTCGT
GmZTL4-R	GTAGTCTCACTGCCCCAAGC
GmFKF1-F	GCATAAAACCAGACCCTCTTCTCTC
GmFKF1-R	CAATCATGTAATAGTGGCTCATATAC
GmFKF2-F	AAAGATTTTGGGTGGGTTTCC
GmFKF2-R	GTGTAATAGACGGTGGAGGAG
GmGI1-F	CTCGAGATGCATTTGGTTGTCTTC

GmGI1-R	CCAATACATCACCAACAATTAATC
GmGI2-F	GTAAACCTTCTCATTCTGCTAG
GmGI2-R	CATTGCTTGAAGTCGTGTTTGGG
GmGI3-F	CTGGATAGAACAGACTTGTACAG
GmGI3-R	GGAAATCCTGCCTGAACATAC
GmELF3a-F	GCAGTTTGGCTCCTCAGTTG
GmELF3a-R	CACTCCACACTCCAGCTGTA
GmELF3b-F	TGTACACTTGCTCGGGGATT
GmELF3b-R	ATCAACAGGAAGGGAGCAGG
GmELF4a-F	TCGGTGCTGGATCGGAAC
GmELF4a-R	ACGTTGGTGAAATTGGTGTTG
GmELF4b-F	CAACAACCTTGAGCACGACGA
GmELF4b-R	CTTGTTCAGGGTGACCCACG
GmELF4c-F	TCCTGACAACATGGCGAAGA
GmELF4c-R	TCCGCCTCATCTTCATCCTC
GmPILa-F	GCCATCCCCAACGAAGACCAC
GmPILa-R	GGAGATCGCAGTCTGGTGGTTG
GmPILb-F	ACGGCGCATCACATGAATCCTC
GmPILb-R	TGCTGGCATGGGTGGTATTTGG
GmPILc-F	ACATCAACCTGGCGGAAAACCC
GmPILc-R	TGGCTAGTGGGGAAGGGAAACC
GmFTL3-F	GATGGGGATTCATCGTTTGGTG
GmFTL3-R	TTAGTATAACCTCCTTCCACCAG
GmFTL4-F	TCGCCCTAGGGTTACTGTTG
GmFTL4-R	AGTGTCTCTGCCCAATTGCT
GmLUXa-F	GATGGAGTGGGAGATGGGTC
GmLUXa-R	GTTGGATGAGAAGGCCTGGT
GmLUXb-F	CCAACTGCCAACGATCTCAC
GmLUXb-R	TTGTTGGAGGAGGAGAAGGC
Tubllin- F	TCTTGGACAACGAAGCCATCT
Tubllin- R	GGTGAGGGACGAAATGATCT
